# A Review of the Pharmacological Properties of Psoralen

**DOI:** 10.3389/fphar.2020.571535

**Published:** 2020-09-04

**Authors:** Yali Ren, Xiaominting Song, Lu Tan, Chuanjie Guo, Miao Wang, Hui Liu, Zhixing Cao, Yuzhi Li, Cheng Peng

**Affiliations:** ^1^School of Pharmacy, Chengdu University of Traditional Chinese Medicine, Key Laboratory of Standardization for Chinese Herbal Medicine, Ministry of Education, National Key Laboratory Breeding Base of Systematic Research, Development and Utilization of Chinese Medicine Resources, Chengdu, China; ^2^Jiangsu Key Laboratory of Drug Discovery for Metabolic Disease, China, Pharmaceutical University, Nanjing, China

**Keywords:** psoralen, osteoporosis, tumor, inflammatory, Severe Acute Respiratory Syndrome Coronavirus 2

## Abstract

Psoralen is the principal bioactive component in the dried fruits of *Cullen corylifolium* (L.) Medik (syn. *Psoralea corylifolia* L), termed “Buguzhi” in traditional Chinese medicine (TCM). Recent studies have demonstrated that psoralen displays multiple bioactive properties, beneficial for the treatment of osteoporosis, tumors, viruses, bacteria, and inflammation. The present review focuses on the research evidence relating to the properties of psoralen gathered over recent years. Firstly, multiple studies have demonstrated that psoralen exerts strong anti-osteoporotic effects *via* regulation of osteoblast/osteoclast/chondrocyte differentiation or activation due to the participation in multiple molecular mechanisms of the wnt/β-catenin, bone morphogenetic protein (BMP), inositol-requiring enzyme 1 (IRE1)/apoptosis signaling kinase 1 (ASK1)/c-jun N-terminal kinase (JNK) and the Protein Kinase B(AKT)/activator protein-1 (AP-1) axis, and the expression of miR-488, peroxisome proliferators-activated receptor-gamma (PPARγ), and matrix metalloproteinases (MMPs). In addition, the antitumor properties of psoralen are associated with the induction of ER stress-related cell death *via* enhancement of PERK: Pancreatic Endoplasmic Reticulum Kinase (PERK)/activating transcription factor (ATF), 78kD glucose-regulated protein (GRP78)/C/EBP homologous protein (CHOP), and 94kD glucose-regulated protein (GRP94)/CHOP signaling, and inhibition of P-glycoprotein (P-gp) or ATPase that overcomes multidrug resistance. Furthermore, multiple articles have shown that the antibacterial, anti-inflammatory and neuroprotective effects of psoralen are a result of its interaction with viral polymerase (Pol), destroying the formation of biofilm, and regulating the activation of tumor necrosis factor alpha (TNF-α), transforming growth factor beta (TGF-β), interleukin 4/5/6/8/12/13 (IL-4/5/6/8/12/13), GATA-3, acetylcholinesterase (AChE), and the hypothalamic-pituitary-adrenal (HPA) axis. Finally, the toxic effects and mechanisms of action of psoralen have also been reviewed.

## Introduction

The dried fruits of *Cullen corylifolium* (L.) Medik, termed “Buguzhi” in China, described in “Leigong’s Treatise on the Preparation of drugs”, have been widely used as medicinal herbs for 1,000 years and are listed in the Chinese Pharmacopoeia (Li K. et al., 2019). According to the theory of traditional Chinese medicine, it is used for nourishing the kidneys and tonic yang, warming the spleen, relieving diarrhea, and supporting qi and asthma, caused by osteopathy, neoplastic infection and inflammation. Modern pharmacology research indicates that the principal components of *Cullen corylifolium* (L.) Medik are coumarins, flavones, and terpene phenols, which have anti-osteoporotic, antitumor, antibacterial, antioxidant, photosensitive, antidepressant, and other pharmacological properties.

**Figure 1:**
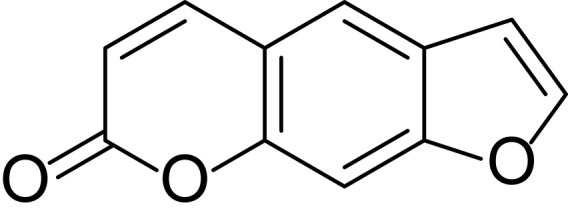
Chemical structure of Psoralen.

Psoralen is a principal bioactive component of *Cullen corylifolium*(L.)Medik, and is also found in many vegetables and fruits, such as *Apium graveolens* L and *Ficus carica* L. As shown in **Figure 1**, psoralen is a tricyclic coumarin-like aromatic compound, the molecular structure of which is 7H-Furo[3, 2-g]benzopyran-7-one (molecular weight: 186.16; molecular formula: C_3_H_6_O_3_) (Sui et al., 2020). Psoralen is widely used as a quality control component in herbal formulae such as Sishen Wan, Yaotong Pian, and Wenweishu Jiaonang (Ji et al., 2015; Hai et al., 2017), and combined with ultraviolet-A light for the treatment of psoriasis, vitiligo, and eczema (Madigan and Lim, 2016). This article will review the pharmacological properties of psoralen, including its anti-osteoporosis, anti-tumor, anti-viral, antibacterial and anti-inflammatory effects, and its toxicity. Psoralen is a natural product of considerable interest that has attracted much attention in the research community due to having wide distribution across a variety of plant species, combined with its excellent potential as a pharmaceutical agent. This suggests that psoralen has broad prospects in therapeutic applications. Multiple studies have focused on its pharmacological effects, but a comprehensive and systematic review of the literature has not been published. This review aimed to collate recent studies on psoralen so as to provide an exhaustive reference for researchers.

## Clinical Research

*Cullen corylifolium* (L.) Medik (Buguzhi) is widely used as a traditional Chinese medicine. It is not only used in the formulae of a variety of traditional Chinese medicines to treat various diseases but it can also be combined with ultraviolet light to treat a number of skin diseases. Zhuanggu Guanjie Wan is formulated from psoralen as the main raw material and was recorded in the Chinese Pharmacopoeia in 2015. It can be used to treat osteoarthritis and is widely used in clinic (Zeng et al., 2006). In addition, Yifei Qinghua Gao is used clinically as an adjuvant treatment for advanced lung cancer (Liu and Qi, 2016; Li D. R. et al., 2017), and Gunben Kechuan Pian is used to treat chronic bronchitis, emphysema, and bronchial asthma (Sun et al., 2009). Baidianfeng Jiaonang is administered for the treatment of vitiligo (Guo et al., 2016). Psoralen, the principal active ingredient of Psoralea corylifolia L (Zhang et al., 2016), plays an important role in the treatment of osteoporosis, tumors, inflammation, *etc*. Therefore, research on psoralen is beneficial for the more widespread treatment of diseases such as osteoporosis, tumors, and inflammation.

## Anti-Osteoporotic Effects of Psoralen

### Promotion of Osteoblast Proliferation and Differentiation

Osteoblasts can effectively inhibit the occurrence and development of osteoporosis by secreting type I collagen (Col-I) and supporting its calcification. Promotion of osteoblast proliferation and differentiation is an effective treatment strategy for osteoporosis. Glucose transporter 3 (GLUT3) is a transporter with high affinity for glucose, important for osteoblast proliferation (Masin et al., 2014). The crucial transcription factor runt-related transcription factor 2 (Runx2) is involved in osteoblast differentiation (Li et al., 2018). Alkaline phosphatase (ALP) is a calcium-binding transporter located in the cell membrane which promotes cell maturation and calcification. Studies have demonstrated that psoralen increases the gene expression levels of osteoblast-specific markers such as GLUT3, Runx2, Col-I, osteocalcin, bone sialoprotein, and osterix, in addition to enhancing the activity of ALP, increasing colony formation in fibroblasts and promoting osteoblast differentiation (Tang et al., 2011; Yang et al., 2012; Li F. et al., 2017; Zhang et al., 2019).

BMPs serve vital roles in the differentiation of osteoblasts. BMP-2/4 promote the expression of ALP and Col-I (Soundharrajan et al., 2018) and bind to the cell surface receptors on bone cells, inducing the phosphorylation of Smad1/5/8. Subsequently, Smad4 combines with phosphorylated Smad1/5/8 and is transported to the nucleus where it activates bone-specific gene transcription, and accordingly stimulates bone formation (Abdallah and Ali, 2019; Jungbluth et al., 2019). It has been further established that psoralen enhances the gene expression levels of BMP-2/4, increases phospho-Smad1/5/8 protein expression and induces the activity of BMP reporter 12xSBE-OC-Luc. It also enhances the expression of Osterix (OSX), a direct target gene of BMP signaling. The deletion of BMP-2/4 genes eliminates stimulation by psoralen of osteoblast marker gene expression (Tang et al., 2011). These results suggest that psoralen promotes osteoblast differentiation through activation of BMP signaling, and may represent a potential anabolic agent for the treatment of osteoporosis and other bone-loss related diseases.

Mitogen-Activated Protein Kinases (MAPKs) play important roles in regulating cell growth, differentiation, and morphogenesis in a variety of tissues (Yasoda et al., 2004). It has been confirmed that MAPK signaling pathways participate in osteoblast proliferation (Hou et al., 2019). One study confirmed that psoralen stimulates osteoblast proliferation through the extracellular signal-regulated kinase (ERK) / MAPK, JNK / MAPK, p38 / MAPK, and Nuclear factor-κB (NF-κB) pathways (Li F. et al., 2017). Similar to BMP, TGF-β is also a membrane protein upstream of ERK, JNK, and P38/MAPK. It is controlled by a variety of factors such as the ubiquitin proteasome system, epigenetic factors, and microRNA (Wu et al., 2016). Therefore, we hypothesize that psoralen may play a role in bone development through TGF-β signaling.

Distal-Less Homeobox 5 (DLX5) serves a vital role in bone development and healing. Osteopontin (OPN) is generally secreted by osteoclasts, osteocytes and osteoblasts, and is involved in the process of absorption and mineralization of bone matrix (Li et al., 2018). In human periodontal ligament cells (HPDLCs), psoralen markedly increases the protein expression levels of the osteogenic proteins DLX5, Runx2 and OPN and facilitates bone formation in periodontal tissue (Li et al., 2018).

IRE1 can coactivate ASK1 with TNF-receptor-associated factor 2 (TRAF2) to form the IRE1/TRAF2/ASK1 trimer. ASK1 is a key regulator of cell apoptosis and can promote the phosphorylation of its downstream JNK. Phosphorylated JNK can activate the genes of pro-apoptotic proteins thereby inducing apoptosis (Chen et al., 2013a; Choi et al., 2020). Chen et al. found that psoralen inhibits the expression of upstream IRE1 and reduces the phosphorylation of ASK1 and JNK, promoting the proliferation of osteoblasts and blocking the apoptosis of osteoblasts. In addition, they also found that the expression of B-cell lymphoma-2 (Bcl-2), which inhibits apoptosis, increases, while Bax, which promotes apoptosis, decreases in psoralen-treated osteoblasts (Chen et al., 2017a).

Psoralen can promote the proliferation and differentiation of osteoblasts through BMP, MAPK, and IRE1 signaling, promoting bone growth and providing a theoretical basis for the treatment of certain bone-related diseases.

However, the effect of psoralen on the phosphorylation of ASK1, JNK, and MAPK P38 in osteoblasts and osteoclasts remains controversial and requires further study (Li F. et al., 2017; Chen et al., 2017a; Chai et al., 2018; Zhang et al., 2019).

### Inhibition of Osteoclast Differentiation and Activation

The principal nuclear factor-κB ligand (RANKL) signaling cascade constituted by transcription factors I kappaB (I-КB), NF-КB, AP-1, phosphorylated ERK and AKT are the downstream signaling pathways of osteoclast proliferation and differentiation (Lee et al., 2005). Chai et al. reported that psoralen was able to significantly reduce the expression of phosphorylated JNK in osteoclasts. Finally, It has been suggested that psoralen ameliorates osteoclast differentiation and bone resorption through inhibition of AP-1 and AKT pathway activation *in vitro* (Chai et al., 2018).

### Effects on Osteoblasts and Osteoclasts

Osteoprotegerin (OPG) and RANKL can regulate bone remodeling in bone metastasis. OPG can down-regulate RANKL signaling and inhibit the differentiation and activation of osteoclasts by competitively binding RANK and up-regulating the expression of OPG (Lacey et al., 2012). It has been found that the expression of IL-8, macrophage colony-stimulating factor (M-CSF), and parathyroid hormone-related protein (PTHrP) increases in breast cancer cells that have migrated to bone (Hsu et al., 2014). Wu et al. reported that the expression of IL-8, M-CSF, PTHrP, and RANKL decreased but OPG expression increased in osteopathy after treatment with psoralen (Wu et al., 2013). These observations indicate that psoralen can inhibit interaction among osteoclasts, osteoblasts, and cancer cells in tumor-bearing mice, and can significantly reduce the burden of bone metastasis due to breast cancer in mice. Psoralen may be an essential regulator of osteoblast and osteoclast function in tumor-bearing mice by inhibition of the growth of breast cancer cells in the bone microenvironment. Therefore, it may represent a potential bone-modifying agent in the treatment of bone metastasis. However, further research is required to elucidate the role psoralen has in modifying the function of these cell types and in the microenvironment used in this model.

Following a fracture, osteoclasts resorb the dead ends of the bone, creating space for new bone formation (Gomez-Barrena et al., 2015; Hankenson et al., 2015). Osteoblasts then migrate into the site to secrete new bone matrix (Zakłos-Szyda et al., 2020). After freshly formed bone combines with the fractured bone ends, osteoclast-induced bone resorption and osteoblast-guided bone formation continue the bone remodeling process. Research has demonstrated that there are considerable numbers of interactions between osteoblasts and osteoclasts, which synergistically promote, then inhibit fracture healing (Aghajanian and Mohan, 2018). Based on the complementary actions of osteoclasts and osteoblasts, Zhang et al. demonstrated that psoralen is able to promote osteoclast differentiation by activating ERK signals which could also promote osteoblast differentiation, That is, psoralen can enhance the viability of osteoblasts and osteoclasts by activating the ERK signaling pathway, thereby promoting fracture healing. In addition, osteoclast and osteoblast differentiation induced by psoralen can be inhibited by a specific inhibitor of phosphorylated ERK (Zhang et al., 2019).

### Protecting Chondrocytes

A number of molecular components including Wnt-4, glycogen synthetase kinase-3β (GSK-3β), β–Catenin and frizzled-2 represent the constituent parts of the Wnt/β-catenin signaling pathway and have been reported to have an association with osteoarthritis (OA) (Clevers and Nusse, 2012; Lietman et al., 2018). Zheng et al. observed that the expression levels of cyclin D1, Wnt-4, β-Catenin and frizzled-2 in chondrocytes treated with psoralen clearly increased, while those of GSK-3β were down-regulated. Cyclin D1 is a factor crucial to the cell cycle. Additional studies have confirmed that psoralen increases the expression of cyclin D1 by regulating the Wnt/β-catenin signaling pathway, increasing the proliferation of chondrocytes (Zheng et al., 2017).

MMPs, and MMP-13 in particular, maintain the metabolic balance of extracellular matrix (ECM) in cartilage. Other members of the MMP family can be activated when MMP-1/2/3 are highly expressed (Jabłońska-Trypuć et al., 2016). The mutual activation capability represents a complex protease network in synovial fluid, which may inhibit the regeneration of damaged tissues (Zhang et al., 2014). Wang et al. demonstrated that psoralen can down-regulate MMP-1/2/3/9/12/13 gene expression and inhibit the synthesis of MMP-13 protein, indicating that it can potentially stimulate chondrocyte proliferation and increase cartilage-specific gene expression. This ultimately protects chondrocytes from the abnormal gene expression induced by TNF-α Wang C. et al., 2019. Inflammation is a typical biological feature of OA, which manifests as the secretion of MMPs or inflammatory mediators leading to the degradation of cartilage. The dual functions of Psoralen in inhibiting inflammation and activating and protecting chondrocyte physiology represents an alternative agent for the treatment of OA.

Psoralen also up-regulates the expression of Col-II, the principal component of cartilage matrix, promoting the synthesis of cartilaginous ECM and inhibiting the degradation of cartilage matrix, which protects the viability of chondrocytes *in situ*, delaying the erosion of the cartilage surface, and ultimately preventing the degradation of articular cartilage by monosodium iodoacetate (MIA)-induced OA (Xu et al., 2015; Zheng et al., 2017; Wang C. et al., 2019). Xu et al. demonstrated that psoralen increased the expression levels of Sox-9 and proteoglycan, promoting the synthesis of glycosaminoglycan (GAG) and activating chondrocytes *in vitro* (Xu et al., 2015), suggesting it has potential as a treatment for OA or osteoporosis (OP). However, there are certain limitations. It is unclear whether psoralen exhibits differences in its effects n other types of OA.

### Other Actions

PPARγ is mostly involved in the regulation of fat. Excessive differentiation of adipose tissue leads to sparseness of bone trabeculae, proliferation of bone fat cells, resulting in osteoporosis, and even slight fractures in severe cases (Corder et al., 2016; Horowitz and Tommasini, 2018). Li et al. have demonstrated that psoralen can improve pathological changes in steroid-induced avascular necrosis of femoral heads (SANFHs). Psoralen reduced the protein expression levels of PPARγ and increased the expression of osteocalcin (Li H. et al., 2019). The experiment demonstrated that psoralen was able to reduce adipogenesis in bone marrow, promote calcium deposition, and prevent osteoporosis, thus having a positive effect on the rehabilitation of patients with ANFH.

Huang et al. found that miR-488 was downregulated in bone marrow mesenchymal stem cells (BMSCs) following treatment with psoralen. Runx2 was found to be a potential target of miR-488 (Huang et al., 2019). It has been suggested that the targeting of Runx2 by miR-488 may participate in osteogenic differentiation after treatment with psoralen. miR-488 / Runx2 represents an association that has so far not been used for the treatment of osteoporosis. Intervention using miR-488 to target Runx2 may represent a mechanism-based treatment strategy for osteoporosis. Yang et al. reported that Psoralen improved bone mass indicators by elevating trabecular thickness and reducing trabecular space (Yang et al., 2012). Wong et al. suggested that Psoralen in collagen matrix enhanced new local bone formation and could be used for bone transplantation or induction of bone formation (Wong and Rabie, 2011).

miRNAs, such as miR-182-5p, hsa-miR-205-5p, miR-370, and miR-140-5p, can affect the process of bone resorption and formation by regulating MAPK, BMP, RUNX2 *etc* (Itoh et al., 2012; Hwang et al., 2014; Yang and Fang, 2016; Suttamanatwong, 2017; Pan et al., 2018). The therapeutic potential of miR-138, miR-338-3p and miR-188 has been confirmed in OP (Suttamanatwong, 2017). In addition, it has been found that the regulation of upstream TLR4 and TRAF6 affects the NF-κB and MAPK signaling pathways, and Ca^2+^ influx, ultimately inhibiting osteoclast activation (He, 2013).

We, therefore, hypothesize that psoralen may operate by acting on miRNAs, TLR4 or TRAF6 to regulate various signaling molecules in the treatment of various bone-related diseases. However, the specific mechanisms require further study.

**Figure 2:**
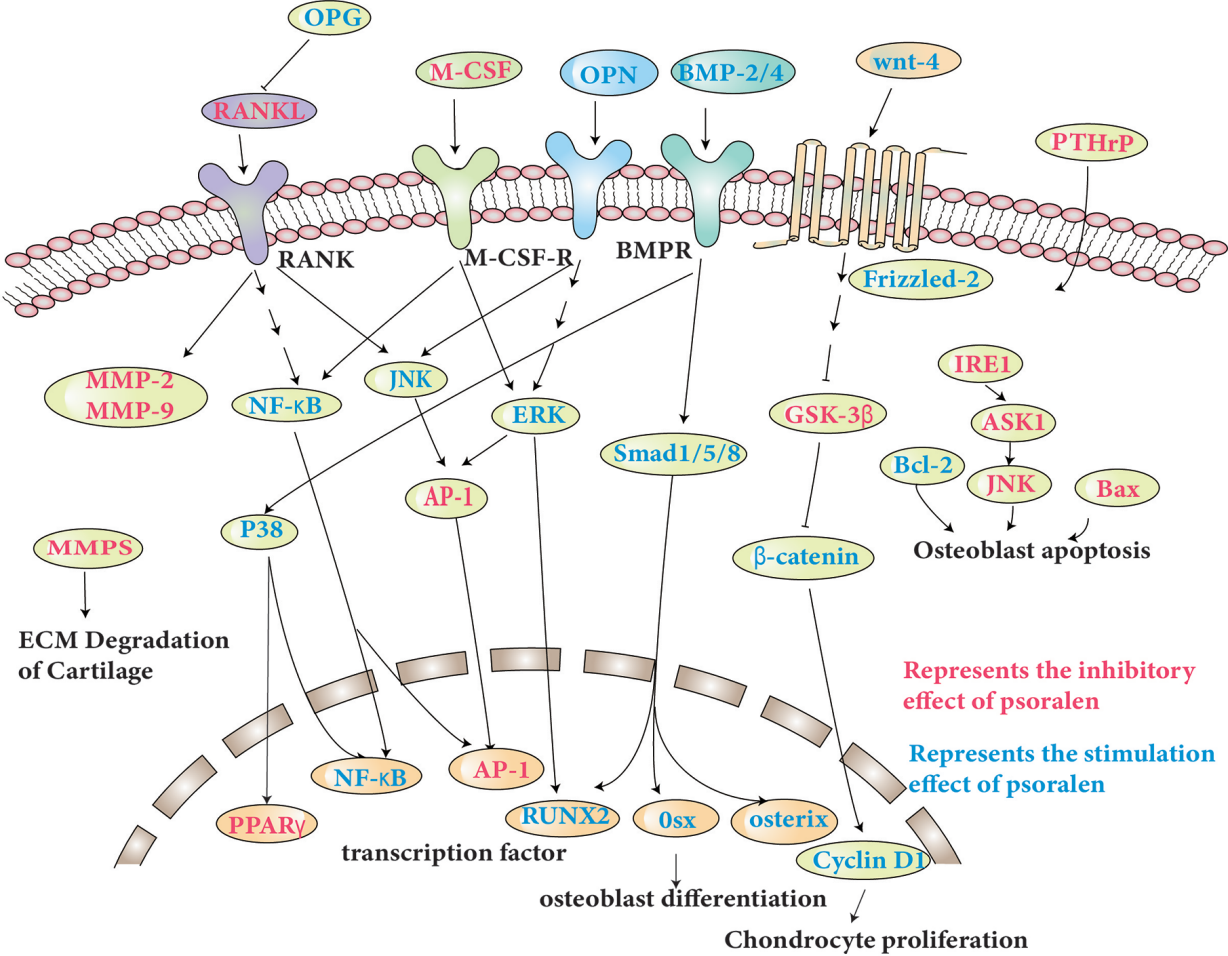
Molecular pathways involved in the anti-osteoporosis actions of psoralen.

The molecular pathways specifically involved are discussed and shown in **Table 1**, and some of the pathways shown in **Figure 2**.

**Table 1:** Pharmacological molecular mechanisms of psoralen.

Models	Ususal doses/concentrations	Molecular mechanisms	References
1. The anti-osteoporosis effect of psoralen			
Primary mouse calvarial osteoblasts	10, 100 μM	Activated **BMP** signaling pathway	Tang et al. (2011)
Human periodontal ligament cells (HPDLCs)	1.6–12.5 µg/ml	Upregulated the expression of osteogenic protein **Runx2, DLX5** and **OPN**	Li et al. (2018)
hFOB1.19 cells	5, 10, 15, 20 µM	Stimulated **NF-κB-MAPK** signaling pathway	Li F. et al. (2017)
Osteoporotic osteoblasts	16 μmol/L	Suppressed the **IRE1/ ASK1/ JNK** pathway.	Chen et al. (2017a)
Mature osteoclasts	0.05, 0.1 μM	Inhibited the activation of **Akt** and **AP-1** pathway	Chai et al. (2018)
Tumor-bearing female nude mice	17.5 mg/kg	Inhibited the interaction among cancer cells, osteoblasts, and osteoclasts, and reduced the burden of bone metastasis of breast cancer in mice	Wu et al. (2013)
Murine osteoblastic MC3T3-E1 cells osteoclasts	2.5, 5, 10, 20, 40 μM	Activated **ERK** signaling pathway	Zhang et al. (2019)
Chondrocytes	10^-8^, 10^-7^, 10^-6^, 10^-5^, 10^-4^ mol/l	Regulated the **Wnt/β-catenin** signaling pathway	Zheng et al. (2017)
Rat chondrocytes	1, 10, 20 µM	Inhibited the expression of **MMPs**	Wang C. et al. (2019)
Articular chondrocytes	1, 10, 100 μM	Activated chondrocytes	Xu et al. (2015)
SANFH rabbit	35 mg/kg	Reduced the expression of **PPARγ**, increased osteocalcin expression	Li H. et al. (2019)
BMSCs	20 µg/ml	Reduced the expression of **miR -488**	Huang et al. (2019)
Female Sprague-Dawley rats	4 mg/ml	Elevated trabecular thickness and reduced trabecular space	Yang et al. (2012)
New Zealand White rabbits	0.25 mg/ml	Enhanced new bone formation	Wong and Rabie (2011)
2. The anti-tumor effect of psoralen			
SMMC-7721 cell	40 μM	Enhanced the expression of **GRP78, GRP94, DDIT3, ATF4, XBP1, GADD34, GDF15** and **IRE1α**, activated **ER stress** signaling pathway	Wang X. et al. (2019)
HepG2 cell	50, 100, 200, 400 μmol/L	Activated **ER-stress** related pathways, activated **Caspase-3/8** and up -regulated the expression of **CHOP** and **Bax**	Yu et al. (2020)
HepG2 cell	150, 300, 450 μM	Inhibited viability	Zhou et al. (2018)
SMMC-7721 cell	10, 30, 50, 100 μg/ml	Up-regulated expression of **Bax, Caspase-3, p53** and reduced protein expression	Jiang and Xiong (2014)
KB, KBv200, K562, K562/ADM cells	50 μg/ml	Induced apoptosis	Wang et al. (2011)
MCF-7 MDA-MB-231cells	8 μg/ml 12 μg/ml	Regulated **Wnt/β-catenin** signaling pathway	Wang et al. (2018)
SMMC-7721 cell	40 μM	Blocked the cell cycle in **G1 phase**	Wang X. et al. (2019)
MCF-7/ADR cell	21.5, 43.0, 64.5, 86.0, 107.5 µM	Arrested the **G0/G1 phase**	Wang X. H. et al. (2016)
MCF-7 cell	10^-7^, 10^-6^, 10^-5^ mol/l	As an estrogen receptor agonist	Xin et al. (2010)
A549/D16 cell	5, 10, 20 μM	Inhibited the activity of **ABCB1** promoter	Hsieh et al. (2014)
MCF-7/ADR cell	8 µg/ml	Inhibited efflux function of **P-gp** transporter	Jiang et al. (2016)
MCF-7/ADR cell	43 µM	Inhibited the activity of P-gp protein dependent **ATPase**	Wang X. H. et al. (2016)
Molecular docking	0.03, 0.1 mM	Inhibited **NF-κB/DNA** interactions	Marzaro et al. (2013)
MCF-7/ADR cell	43 µM	Repressed the activation of **NF-κB p65**	Wang X. H. et al. (2016)
HBL-100 cell	50, 100 μM	Protective effect	Du J. et al. (2019)
MCF-7 and MCF-7/ADR cells	50μM	Down-regulated **MMP1**, **HSD17B6, INHBA** and **CD63** Protein levels, overexpressed SESN3 gene expression	Wang X. et al. (2016)
JB6 cell	14.8, 15.6, 17.1 µg/ml	Induced QR activity, inhibited ODC activity	Lee et al. (2011)
Osteosarcoma SD rat	320μg/(kg·d), 1600 μg/(kg·d)	Reduced the serum **ALP** level	Lu et al. (2014)
3. The antiviral and antibacterial effect of psoralen			
MHV-68	10 μg/ml	Antiviral activity	Cho et al. (2013)
HBV	10 μg/ml	Interacted with **HBV pol**	Parvez et al. (2019)
Orthomyxoviruses CCHFV LASV	10 μg/ml	Keep the particles and RNA intact and non-infectious	Schneider et al. (2015)
DENV-1	10 μg/ml.	Retained its three-dimensional structure	Maves et al. (2010); Maves et al. (2011)
A/PR/8/34 H1N1 virus	0.2 µg/ml	Inhibited the replication	Choi et al. (2017)
H*_37_*RV	220.4 µg/ml	Antibacterial effect	Chiang et al. (2010)
P. cinnamomi mycelia	100, 150 mg/L	Inhibited the growth	Villegas et al. (2015)
P.gingivalis	6.2 µg/ml	Inhibited the formation of biofilm, eliminated the established biofilm, reduced the viability of biofilm	Li et al. (2018)
4. The anti-inflammatory effect of psoralen			
human neutrophils	10.9 μM	Inhibited superoxide anion generation	Chen C. H. et al. (2017)
Raw 264.7 cells	2.5, 5, 7.5 μg/ml	Inhibited the expression of **IL-6** and **TNF-a**	Chen et al. (2013b)
D10 cells stimulated by concanavalin A (Con A)	2, 4, 8×10^-2^ mM	Inhibited the expression of Th2 cytokines **IL-4/5/13** and Th2 transcription factor **GATA-3**	Jin et al. (2014)
THP-1 cell	1.56, 3.13, 6.25, 12.5 µg/ml	Released the expression of **IL-1β** and **IL-8**	Li et al. (2018)
Murine fibroblast NIH3T3 cells	5, 10, 20, 40 μM	Reduced the expression of **TNF-α, IL-1β, and TGF-β1**,	Du M. Y. et al. (2019)
TNF – α induced inflammation of synovial cells	1, 10, 20 µM	Down-regulated the expression of **IL-1 β, - 6, - 12**	Wang C. et al. (2019)
HEPG-2 Cell	10, 50, 100, 150, 200 μM	Inhibited **COX** activity	Ai et al. (2019)
5. The effect of psoralen on melanocytes			
Channa punctatus and Bufo melanostitus	1, 2, 4, 8, 16, 32, 64×10^-7^ g/ml	Stimulated of cholinergic receptors	Meitei and Ali (2012). Sultan and Ali (2011)
Melanocytes	0.5, 1.0, 3.0, 5.0, 7.0, 10 μg/ml	Reduced the survival rate	Quintão et al. (2019))
6.The neuroprotection properties of psoralen			
Adult neural stem cells	100 nM	Increased the expression of **GFAP**, reduced the expression of **TuJ1**,	Ning et al. (2013)
Scopolamine-induced amnesia rats	0.1, 0.3 mg/kg	inhibited **AChE** activity	Wu et al. (2007)
Adult male Wistar rats	100, 200 μg/ml	Connected to the residues of the **AChE** binding site by π-π conjugate and hydrogen bonding	Somani et al. (2015)
The FST-treated mice	10, 20, 40 mg/kg	Attenuated alterations in **5-HT** and **5-HIAA** levels	Xu et al. (2008)
7. Pharmacological properties of psoralen on muscle atrophy and fibrosis			
The myoblast (C2C12) cell line	20, 40, 60, 80, 100, 120 μM	Attenuated the expression of **MuRF1, MAFbx, trim62** and **GDF15** and **miR-675-5P**	Lin et al. (2019)
bleomycin -induced mouse	5 mg/kg/day	Reversed the expression of **α - SMA**	Du M. Y. et al. (2019)

## The Anti-Tumor Effects of Psoralen

### Increasing Endoplasmic Reticulum (ER) Stress-Dependent Apoptosis

The endoplasmic reticulum is an intracellular membrane organelle whose principal function is the synthesis and secretion of membrane proteins, their correct folding, and the storage of Ca^2+^ (Cubillos-Ruiz et al., 2017). Due to the influence of external or internal factors, misfolded and unfolded proteins accumulate in the endoplasmic reticulum, causing the balance of Ca^2+^ to become disordered which leads to ER stress. Tumor cells display strong growth and metabolism, although adverse conditions can result in protein misfolding and accumulation. Tumors can adaptively regulate protein folding through ER stress. The activation of a variety of ER stress sensors, such as GRP78, GRP94, Activating Transcription Factor 6 (ATF6) and PERK, in addition to other related regulatory proteins, has been shown to confer increased tumorigenicity, drug resistance and metastasis on malignant cells. However, when these adaptive mechanisms cannot resolve protein-folding defects, cells enter apoptosis (Wang and Kaufman, 2014; Cubillos-Ruiz et al., 2017).

GRP78, also termed Bip, is considered to be the homeostasis receptor of the endoplasmic reticulum. In physiological conditions, PERK, IRE1 and ATF6 remain inactive due to their occupation of GRP78 (Almanza et al., 2019). In conditions of stress in the endoplasmic reticulum, large unfolded or misfolded proteins cleave GRP78 which is normally combined with three types of response proteins, causing them to become exposed then activated, resulting in expression of CHOP. However, high CHOP expression causes the loss of Ca^2+^ from the endoplasmic reticulum, causing increased mitochondrial permeability and apoptosis (Szalai et al., 2018).

The phosphorylation of PERK promotes translation of ATF4 and also causes phosphorylation of the catalytic substrate eIF2α which inhibits the synthesis of protein leading to apoptosis following long-term inhibition (Hetz, 2012). ATF4 overexpression activates the CHOP apoptotic pathway, thereby activating GADD34 that results in oxygen-free radical damage (Rosenbaum et al., 2014).

GRP94 is found mostly in the endoplasmic reticulum and represents a crucial factor in the ER stress response. In such a response, GRP94 induces the expression of the CHOP protein, thereby attenuating the expression levels of Bcl-2 and promoting apoptosis (Rosenbaum et al., 2014).

Activated ATF6 promotes the expression of CHOP, which then inhibits the expression of Bcl-2 and causes apoptosis (Xu et al., 2018). In addition, growth differentiation factor 15 (GDF15) can inhibit A549 cell proliferation, invasion, and migration, inducing apoptosis (Duan et al., 2019). Wang et al. found that psoralen enhanced the expression of GRP78, GRP94, CHOP, ATF4, X-Box Binding Protein 1 (XBP1), GADD34, GDF15, and IRE1α. Activated ER stress, causing expansion and dysfunction in the endoplasmic reticulum, was found to inhibit SMMC7721 cell proliferation, thereby resulting in apoptosis of liver cancer cells (Wang X. et al., 2019). Furthermore, Yu et al. found that psoralen markedly increased the expression of ATF6, ATF4, PERK, eukaryotic initiation factor 2 (eIF2α), and GRP78, in addition to enhancing the levels of CHOP, Bax, and phosphorylated JNK. The results illustrate that induced ER stress mediates apoptosis through the PERK/ATF pathways (Yu et al., 2020).

However, psoralen had little effect on the proliferation of HepG2 cells (Wang X. et al., 2019). This is controversial because Zhou et al. demonstrated that psoralen reduced the viability of HepG2 cells, primarily by inhibiting proliferation (Zhou et al., 2018). Moreover, Yu et al. indicated that psoralen activates Caspase-3/8, and up-regulates Bax and CHOP expression, apparently inducing HepG2 cell apoptosis (Yu et al., 2020). Liver damage caused by psoralen is more likely to be related to oxidative stress, mitochondrial damage and endoplasmic reticulum dysfunction. However, the overall mechanism of psoralen-induced liver injury and its relationship with endoplasmic reticulum stress remains to be studied.

In addition, Jiang et al. reported that psoralen can inhibit the viability of SMMC-7721 cells, displaying strong promotion of cell apoptosis through the up-regulation of Bax, p53 and Caspase-3 expression, and reducing Bcl-2 protein expression (Jiang and Xiong, 2014).

Wang et al. found that psoralen displayed dose-dependent anti-tumor activity towards the KB and KBv200 (vincristine resistance subline of KB) carcinoma lines, and K562 and K562/ADM (doxorubicin resistance subline of K562) human erythroleukemia cells, by induction of apoptosis, thus confirming its anti-cancer potential (Wang et al., 2011).

### Blockade of the Cell Cycle

Wnt signaling serves a pivotal role in modulating cancer cell proliferation by regulation of the cell cycle. The classic Wnt/β-catenin pathway is related to the regulation of tumorigenesis through arrest of the cell cycle. When β-catenin is stable, it accumulates in the nucleus and structurally activates target genes related to the cell cycle, such as Fra-1 which can functionally increase cell and vascular invasiveness (Liu et al., 2017; Gao et al., 2017). Wang et al. found that psoralen increased the expression of Axin-2, a negative regulator of the Wnt/β-catenin/TCF signaling pathway, in MCF-7 and MDA-MB-231 cells, and decreased the expression of β-catenin and its downstream target Fra-1. Additional studies confirmed that psoralen induced cell cycle arrest in breast cancer cells by regulation of the Wnt/β-catenin pathway (Wang et al., 2018).

Cyclin D1 and Cyclin E1 play important roles in cell cycle regulation, acting as positive regulators of cyclin-dependent kinase (CDK). Cyclin D1 binds to CDK4/6 and Cyclin E1 binds to CDK2, promoting transition from G1 phase to S phase, resulting in cell division (Ingham and Schwartz, 2017). Wang et al. found that psoralen increased the expression of Cyclin D1 and decreased the expression of Cyclin E1, causing blockade of the cell cycle in G1 phase and inhibiting SMMC7721 cell proliferation (Wang X. et al., 2019). In addition, Wang et al. found that psoralen reversed multidrug resistance (MDR) by arresting cycling at the G0 / G1 phase, rather than by promoting apoptosis (Wang X. H. et al., 2016).

Interestingly, Xin et al. found that psoralen, an estrogen receptor alpha (ERα) agonist, significantly promoted the proliferation of MCF-7 cells (Xin et al., 2010). However, as previously described, psoralen markedly inhibited the proliferation of MCF-7 cells by inducing G0/G1 phase arrest (Wang et al., 2018). Therefore, the effect of psoralen in MCF-7 cells requires additional exploration.

### Reversing Multidrug Resistance

Extensive studies have demonstrated that the principal mechanism of cancer multidrug resistance is *via* the action of the transmembrane drug efflux protein P-gp, encoded by the human ATP−binding cassette subfamily B member 1 (ABCB1) gene. Overexpression of this gene suppresses the effect of cancer chemotherapy (Genovese et al., 2017). Hsieh et al. found that Psoralen inhibited the activity of the ABCB1 promoter, at least partially reduced the expression of ABCB1 at the transcriptional level, and sensitized drug-resistant cells when combined with chemotherapy drugs, resulting in their death (Hsieh et al., 2014). However, Jiang et al. demonstrated that psoralen had no noticeable impact on the expression of P-gp. The effect of psoralen on multidrug resistance may be related to the inhibition of efflux by the P-gp transporter, rather than a reduction in P-gp mRNA or protein expression (Jiang et al., 2016). The activity of P-gp requires energy from ATP hydrolysis.

Similarly, Wang et al. found that psoralen reversed MDR by inhibiting the activity of P-gp protein-dependent ATPase rather than reducing the protein expression of P-gp (Wang X. H. et al., 2016). A detailed understanding of the mechanism by which psoralen inhibits P-gp transport may be important to overcome MDR. Therefore, further studies are required to clarify these mechanisms, especially the effect of P-gp on adenosine triphosphatase activity.

### Inhibition of Epithelial to Mesenchymal Transition

Psoralen has been previously shown to inhibit tumor invasion and migration *via* inhibition of NF-κB / DNA interactions (Marzaro et al., 2013). Wang et al. demonstrated that psoralen inhibited epithelial to mesenchymal transition (EMT) through suppression of NF-κB p65 activation, weakening the migration of MCF-7 / ADR cells (Wang X. H. et al., 2016). Interestingly, Du et al. found that psoralen had a clearly protective effect on HBL-100 cells from the non-malignant human breast epithelial cells line injured by ionizing radiation, but did not protect MCF-7 tumor cells (Du J. et al., 2019). This suggests that healthy cells treated with psoralen would be protected from radiation damage while tumor cells would be selectively removed. This observation also provides a reference for the protection of healthy cells during the treatment of tumors.

### Regulation of Exosome Secretion

The PPAR signaling pathway regulates the synthesis of ceramide levels, and, like p53, ceramides are important regulatory molecules of exosome secretion (Kita et al., 2019; Pavlakis and Stiewe, 2020). Wang et al. found that the MMP1 gene associated with the PPAR signaling pathway was down-regulated in addition to HSD17B6, the gene coding for inhibin beta A (INHBA) protein, following treatment with psoralen. In addition, expression of the exosome marker CD63 also decreased in MCF-7/ADR cells, and Sestrin 3 (SESN3), associated with the p53 signaling pathway, was overexpressed in the presence of psoralen (Wang X. et al., 2016). The results illustrate that psoralen possibly affects exosomes through PPAR and the p53 signaling pathway, resulting in a decrease in the transmission of drug resistance *via* exosomes, and providing a potential novel strategy for defeating drug resistance in breast cancer in the future.

### Other Actions

Lee et al. found that psoralen was able to inhibit carcinogenesis induced by carcinogens, especially during the initiation and promotion stage through the inhibition of ornithine decarboxylase activity and induction of quinone reductase activity. It was also found to suppress murine epidermal JB6 cell tumor development (Lee et al., 2011).

In osteosarcoma, serum ALP activity is increased. Lu et al. found that psoralen significantly decreased the activity of serum ALP in osteosarcoma in nude rats, and exhibited a significant inhibitory effect on osteosarcoma in nude mice (Lu et al., 2014).

In recent years, the concept of the UPR being able to regulate the anti-cancer immune response has emerged (Lhomond et al., 2018). In addition, Th2 cell-related cytokines can activate the IRE1 pathway. Drugs that inhibit IRE1 can block cathepsin secretion and macrophage-mediated cancer cell invasion (Yan et al., 2016). Psoralen can induce endoplasmic reticulum stress and regulate UPR. Therefore, we suspect psoralen may control cancer through an anti-cancer immune response. The specific mechanism requires additional study.

**Figure 3:**
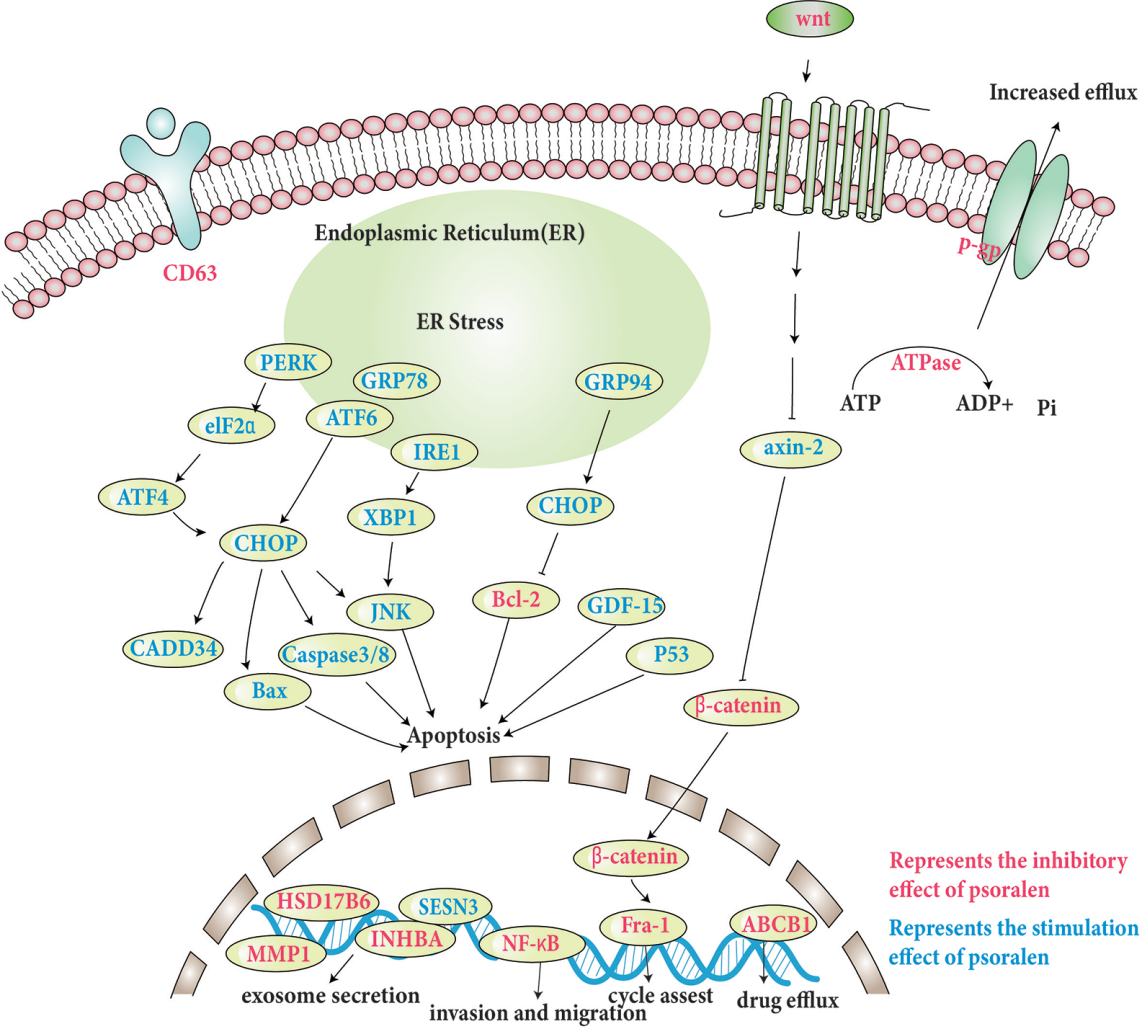
Molecular pathways involved in the anti-tumor effects of psoralen.

The specific molecular pathways involved are discussed and shown in **Table 1**, and some pathways involved are shown in **Figure 3**.

## Antiviral and Antibacterial Effects of Psoralen

### Anti DNA Virus

Gamma herpes viruses such as Kaposi’s sarcoma-associated herpes virus (KSHV) and Epstein–Barr virus (EBV) are important human pathogens responsible for a variety of malignancies (Sethuraman et al., 2018; Jog et al., 2018). Cho et al. demonstrated that psoralen had clear antiviral activity towards murine gamma herpes virus 68 (MHV-68) and exhibited significant inhibition of the lytic cycle of human gamma herpes viruses. Although not all antiviral agents screened from MHV-68 may be effective for both KSHV and EBV (Cho et al., 2013), the results highlight how the MHV-68 replication system can be used to identify candidate antiviral drugs for development against gamma herpes viruses.

The hepatitis B virus (HBV) can cause acute and chronic liver diseases (Teo and Locarnini, 2010). Parvez et al. found that psoralen inhibited HBV replication through HBV Pol, the most important viral protein, representing a potential drug target (Parvez et al., 2019). It has been suggested that psoralen has the potential to be used as a pol / RT inhibitor of HBV.

### Anti-RNA Virus

Recent studies of the antiviral pharmacology of psoralen have shown that it has wide application value in the prevention and treatment of RNA viruses. Firstly, psoralen can inactivate a virus which can then be used in vaccine preparation. Compared with the widely used methods of high temperature or application of toxic chemicals, which denatures or cross-links proteins and nucleic acids, psoralen renders the virus non-infectious, but does not destroy the particles or RNA, but conversely preserves the structures required for genomic analysis, enabling the inactivated virus to be used in vaccine development (Schneider et al., 2015). If this virus inactivation method is to become a generally-accepted method of inactivation, it is necessary to conduct multi-site specificity and drug specificity verification studies, testing the method robustly in a large trial to ensure that the inactivated virus is truly non-infectious.

Dengue viruses are categorized into four distinct serotypes of RNA virus of the Flaviviridae family. Maves et al. found that psoralen was able to inactivate dengue virus type 1 (DENV-1), while retaining the virus’s three-dimensional structure, allowing the production of antibodies with multiple epitopes for priming the immune system. Additional studies have shown that the candidate DENV-1 vaccine raised from inactivated virus using psoralen displayed immunogenicity in mice. The subsequent effects of the candidate vaccine tested on Aotus nancymaae monkeys exhibited a protective effect (Maves et al., 2010; Maves et al., 2011).

Subsequently, Choi et al. found that psoralen significantly inhibited the replication of A/PR/8/34 H1N1 virus and induced macrophages to secrete antiviral cytokines such as interferon-beta (IFN-β). Choi et al., 2017. A recent study found that low concentrations of IFN-β can significantly inhibit Severe Acute Respiratory Syndrome Coronavirus 2 (SARS-CoV-2) infection (Mantlo et al., 2020). This provides a new opportunity to treat Coronavirus Disease 2019 (COVID-19) which is currently causing a global outbreak. SARS-CoV-2, the causative pathogen of COVID-19, is a single-stranded RNA-type β-coronavirus for which there is currently no specific antiviral drug or vaccine (Yang et al., 2020). In addition, psoralen has the characteristics of retaining the three-dimensional structure of the virus, preserving immunogenicity, which is conducive to vaccine production. Therefore, it is anticipated that psoralen could be used as an agent to treat or prevent SARS-CoV-2 and has potential application value in the preparation of related vaccines.

### Antibacterial Properties

Chiang et al. found that psoralen displayed an antibacterial effect on mycobacterium tuberculosis H37Rv (Chiang et al., 2010). Villegas observed that psoralen inhibited the growth of P. cinnamomi mycelia (Villegas et al., 2015), indicating that psoralen has the potential to be a biological pesticide. Li et al. found that psoralen inhibited the formation of biofilms, eliminated established biofilms and reduced their viability (Li et al., 2018). Periodontitis is a chronic inflammatory disease that causes the destruction of periodontal tissues. Psoralen has the dual effects of promoting bone formation and inhibiting major periodontal pathogens, and so can be used for the treatment and prevention of periodontitis. It provides new ideas for the development of new comprehensive therapeutic drugs. The relevant molecular pathways are discussed and shown in **Table 1**.

## Anti-Inflammatory Effects of Psoralen

Human neutrophils play a vital role in the host defense against microorganisms and the pathogenesis of a variety of diseases (Kolaczkowska and Kubes, 2013). Reactive oxygen species (ROS) were thought to be a host defense molecule released by neutrophils to kill foreign pathogens, such as bacteria (Dallenga et al., 2017). Chen et al. found that psoralen strongly inhibited superoxide anion generation in human neutrophils, and thus representing an anti-inflammatory response (Chen C. H. et al., 2017).

The melanocortin 1 receptor (MC1R) gene regulates coat color in mammals (Chen et al., 2017b). Macrophages are important inflammatory cells that participate in the initiation of the inflammatory response, able to secrete TNF-α, IL-6, and other pro-inflammatory mediators, playing a vital role in the development of an inflammatory response (Giavridis et al., 2018). TNF-α and IL-6 can induce the clinical syndromes of atopic dermatitis, including skin allergy syndrome and other immune disorders (Ford, 2016). Chen et al. found that high concentrations psoralen, an MC1R antagonist, preferentially combined with MC1R to cause sustained feedback regulation, promoting the expression of cyclic adenosine monophosphate (cAMP), an important molecule in the MC1R signal transduction pathway. This had the effect of dampening the innate immune-mediated response and expression of TNF-α and IL-6 (Chen et al., 2013b). Taken together, the study demonstrated that MC1R was able to reduce inflammation *in vivo* and *in vitro* and may be an effective target for inhibiting an inflammatory response.

A T helper 2 (Th2) response is the principal pathological mechanism in asthma, in which Th2 cell-derived cytokines are believed to be the driving force for the development of airway hyperresponsiveness, inflammatory cell accumulation and mucus hypersecretion (Holt and Sly, 2007). IL-4 and IL-13 are critical for mucus hypersecretion and IgE production, while IL-5 is essential for the survival, activation, and recruitment of eosinophils (Romagnani, 2000). GATA-3 is a key transcription factor and considered key to the up-regulation of Th2 cytokines (Hosoya et al., 2010). Jin et al. found that psoralen significantly inhibited the expression of the Th2 cytokines IL-4/5/13, and GATA-3 in D10 cells stimulated by concanavalin A, but displayed no inhibition of cell viability (Jin et al., 2014). These findings support the hypothesis that psoralen may be a critical compound for inhibition of a Th2 response in asthma.

Furthermore, Li et al. reported that psoralen significantly decreased the release of IL-1β and IL-8 in THP-1 cells (Li et al., 2018). Du et al. found that psoralen reduced the expression of TGF-β1, IL-1β, and TNF-α in a mouse model of bleomycin-induced primary pulmonary fibrosis (Du M. Y. et al., 2019). Recently, Wang et al. found that psoralen inhibited TNF-α induced inflammation in synovial cells by down-regulation of IL-1β, -6, and -12 gene expression and inhibition of the synthesis of IL-1 β protein (Wang C. et al., 2019).

Cyclooxygenase (COX) is responsible for the formation of prostaglandins. COX-2 is a subtype found in multiple disorders, such as inflammation and many cancers (Limongelli et al., 2010). Ai et al. confirmed that psoralen possessed COX inhibitor activity in HepG2 cells by combining random forest and self-organizing feature map neural networks and through molecular docking analysis (Ai et al., 2019).

It has been reported that the IRE1α-XBP1-cMyc axis has been identified in NK cell immunity, required for the host to resist murine cytomegalovirus (MCMV) infection and cancer (Dong et al., 2019). Psoralen can activate IRE1 and XBP1 and has certain anti-tumor effects. It can achieve anti-viral and anti-inflammatory effects through a variety of means. Therefore, whether psoralen can regulate NK cell immunity through the IRE1α-XBP1-cMyc axis remains to be verified. The relevant molecular pathways are discussed and displayed in **Table 1**.

## Effect of Psoralen on Melanocytes

Melanocytes are large, specialized, flat pigment cells within the skin of vertebrates. They can cause the skin to undergo rapid change in color when exposed to a variety of stimuli and has been used as a unique model to study the complex mechanisms of skin pigmentation (Sultan and Ali, 2011). Vitiligo is a disorder caused by the absence or reduced number of melanocytes, resulting in reduced skin melanin production (Frisoli et al., 2020). Sultan et al. found that psoralen was able to stimulate cholinergic receptors and cause melanocytes to diffuse within the Channa punctatus and Bufo melanostitus (Sultan and Ali, 2011). From these results, Meitei et al. believed that psoralen induced a distinct form of melanin diffusion in reptile skin by mimicking the action of acetylcholine to stimulate cholinergic receptors, an effect antagonized by atropine and scopolamine (Meitei and Ali, 2012). More recently, Quintão et al. found that psoralen reduced the survival rate of melanocytes to a certain extent, but did not significantly change the viability of keratinocytes. Psoralen promoted the production of less melanin than cells stimulated by the melanin-promoting agent 3-isobutyl-1-methylxanthine (Quintão et al., 2019). The relevant molecular pathways are discussed and presented in **Table 1**.

## Neuroprotective Properties of Psoralen

Adult neural stem cells (NSCs) in the mature nervous system are a common source of all nerve cells, including neurons, astrocytes, and oligodendrocytes (Navarro Negredo et al., 2020). Astrocytes excite inhibitory neurons and inhibit the general activities of peripheral neurons that prevent overexcitation of neurons in the nerve ring (Choe et al., 2012). Without the help of astrocytes, neurons cannot produce an enhanced response, the foundation of learning and memory (Frankland and Josselyn, 2020). Ning et al. reported that psoralen was able to inhibit neural stem cell proliferation and self-renewal of nerve cells. In addition, psoralen increased the expression of the astrocyte-specific marker glial fibrillary acidic protein (GFAP), but led to the slightly reduced expression of the neuron-specific marker β-tubulin III (TuJ1). It has been suggested that psoralen can induce astrocyte differentiation. Additional research had found that psoralen specifically regulates the gene expression profile of NSCs (Ning et al., 2013). suggesting that psoralen has the potential to treat neurodegenerative diseases.

Research study suggests that psoralen competitively inhibits AChE activity in a concentration-dependent manner, which therefore activates the central cholinergic neuronal system, and tightly binds the residues at the enzyme binding site by π-π conjugation and hydrogen bonding (Somani et al., 2015), indicating that psoralen may be a potential candidate to inhibit AChE. This should be further explored for clinical applications in Alzheimer’s disease.

Depression is related to behavioral disorders, serotonin, and neuroendocrine dysfunction (Forbes, 2020). Neuroendocrine abnormalities in depression are due to hyperactivity of the HPA axis characterized by excessive secretion of corticotrophin-releasing factor (CRF), which stimulates the release of corticosterone (Horowitz and Zunszain, 2015). CRF is a neuroregulatory factor found in the brain. The transmission of serotonin (5-HT) is regulated by CRF (Marcinkiewcz et al., 2016). 5-HT can be broken down into 5-hydroxyindoleacetic acid (5-HIAA) (Weber et al., 2015). A forced swimming test (FST) can induce abnormalities in the serotonergic and HPA axis (Xiao et al., 2019). Xu et al. found that psoralen increased swimming in a mouse FST, reducing changes in 5-HT and 5-HIAA levels, and attenuating the ratio of 5-HIAA/5-HT in the frontal cortex and hippocampus. Furthermore, psoralen reduced the expression levels of corticosterone and serum CRF and so normalized HPA axis activity. This indicates that psoralen could regulate the serotonin and HPA axis system (Xu et al., 2008), and that psoralen may represent a potential candidate for the treatment of depression. The relevant molecular pathways are discussed and shown in **Table 1**.

## Pharmacological Effects of Psoralen on Muscle Atrophy and Fibrosis

MAFbx, MuRF1, and TRIM62 are members of the E3 ubiquitin ligase family. MAFbx and MuRF1, key regulators of muscle atrophy, are involved in skeletal muscle protein breakdown (Conraads et al., 2010). and have been found to be up-regulated in a variety of muscle atrophy models (Sacheck et al., 2007; Price et al., 2010; Powers et al., 2011). TRIM62 is thought to be involved in the regulation of differentiation, immunity, development and apoptosis, playing a vital role in the Toll like Receptor 4 (TLR4) signaling pathway (Reymond et al., 2001; McNab et al., 2011). Various studies have found that the expression of TRIM62 is increased significantly in the muscles of critically ill patients (Langhans et al., 2014), and that activation of TRIM62 results in persistent muscle inflammation, promoting atrophy in critically ill patients (Uchil et al., 2013). GDF15 is a member of the TGF-β family, also known as macrophage inhibitory factor . Lin et al. previously found that miR-675-5P was significantly up-regulated in patients with muscular dystrophy compared with healthy individuals and that psoralen reduced the cytotoxicity, cell atrophy and apoptosis induced by TNF-α in C2C12 myoblasts. The therapeutic effect of psoralen on muscle atrophy occurs through attenuation of the expression of atrophy marker proteins MuRF1, MAFbx, TRIM62, and GDF15, and miR-675-5P which is expressed in skeletal muscle and up-regulated during myoblast differentiation and muscle regeneration (Lin et al., 2019). These observations provide a theoretical basis for the study of muscular atrophy mechanisms in the future. Du et al. found that psoralen inhibited the proliferation of mouse fibroblasts and had a therapeutic effect in bleomycin-induced primary pulmonary fibrosis. Furthermore, Psoralen partially reversed bleomycin-induced α-smooth muscle actin expression, and collagen synthesis. Additionally, psoralen reduced inflammation in lung parenchyma and increased the survival rate of mice (Du M. Y. et al., 2019). The relevant molecular pathways are discussed and displayed in **Table 1**.

## Safety and Toxicity

**Figure 4:**
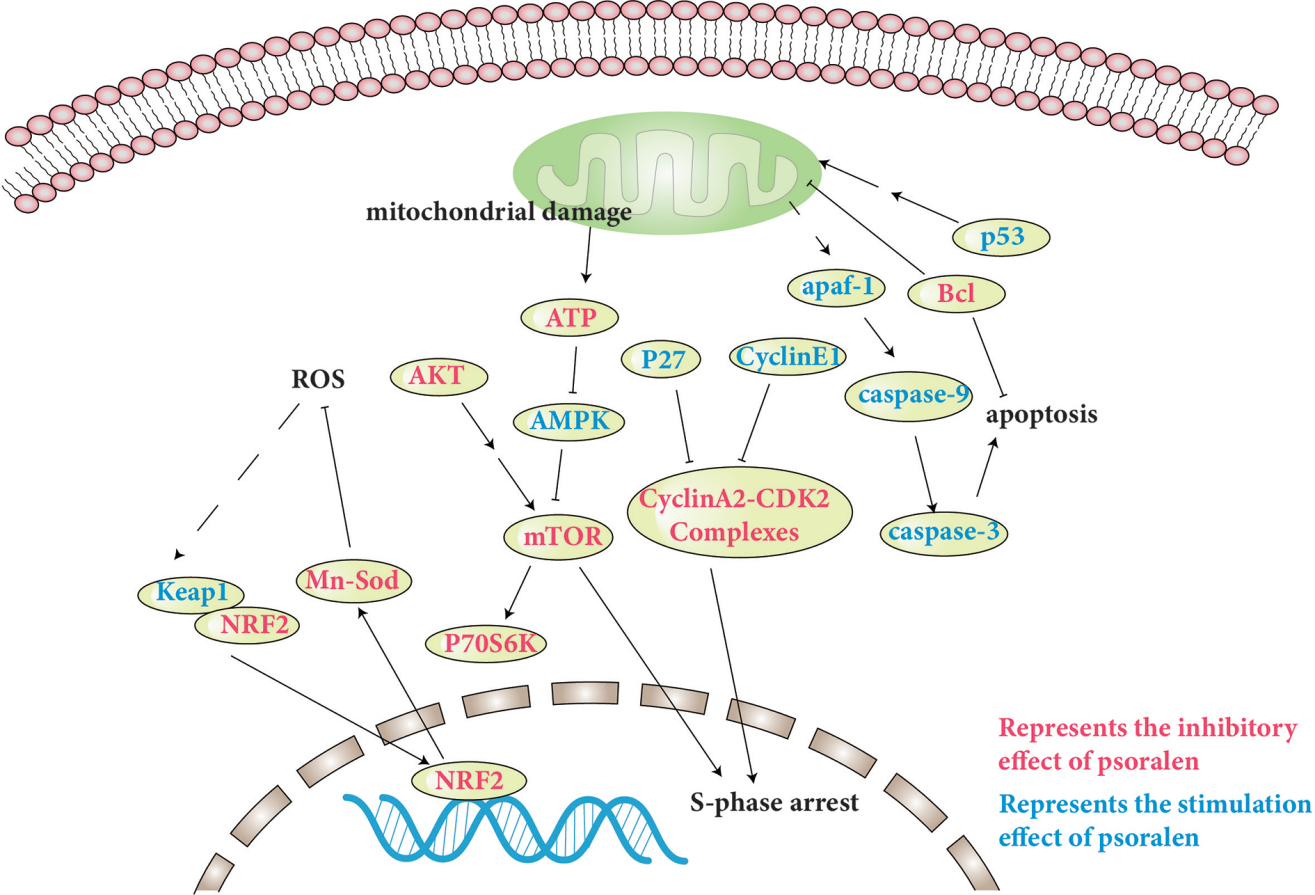
Molecular pathways involved in psoralen safety and toxicity.

The toxicity of psoralen is an issue of wide concern. However, there are few reports of the toxicity of psoralen. In addition, the underlying mechanisms of psoralen-induced toxicity remain unclear. The specific molecular pathways involved are discussed and presented in **Table 2**, and a number of pathways involved are shown in **Figure 4**.

**Table 2:** Molecular mechanisms of the safety and toxicity of psoralen.

Models	Ususal doses/concentrations	Molecular mechanisms	References
Sprague Dawley (SD) rats	60 mg/kg	Regulated the expression of **Cyp1a1, Cyp1a2, Gstm1** and **Akr7a3**	Song et al. (2019)
Male Kun-Ming strain mice	20,40 mg/kg	Inactivated of **CYP2E1**	Wang et al. (2012)
Male SD rats	5 mg/kg	Reversibly inactivated **CYP1A2**	Zhuang et al. (2013)
P4502A6 from the crude insect cell paste	100 µM	Irreversibly inactivated **CYP2A6**	Koenigs and Trager (1998a)
P4502B1	100 µM	Irreversibly inactivated **CYP2B1**	Koenigs and Trager (1998b)
Recombinant human P450 enzymes	40, 80, 120, 160, 200 µM	Irreversibly inactivated **CYP2B6**	Ji et al. (2015)
Human recombinant CYP3A4 enzyme	200 µM	Irreversibly inactivated **CYP3A4**	Liu and Flynn (2015) Hai et al. (2017)
SD rats	60 mg/kg	Increased **ALT** and **AST**, decreased **Glu** and **ALB**, interfered with amino acid metabolism	Zhang et al. (2018)
L02 cell	150, 300, 450 μM	Up-regulated **cyclin E1**, **p27**, down-regulated **cyclin A2**, induced S-phase arrest, inhibited **mTOR** signaling pathway	Zhou et al. (2018)
High glucose-treated mice mesangial MES-13 cells	4 μg/ml	Reduced the expression of cleaved **PARP** and **Bad**, and promoted expression of **phospho-Bad (ser112)** and **Bcl-2**.	Seo et al. (2017)
Zebrafish embryo/larval	10.61µM	Up-regulated **Keap1** expression, down-regulated **Nrf2**, **Mn-Sod hmgcra**, **pparα1** and **fas** expression. increased the expression of **p53, puma, apaf-1, caspase-3/9, caspase-3**, decreased **Bcl-2** expression	Xia et al. (2018)

### Liver Damage

Recently, studies have demonstrated that psoralen exhibits hepatotoxicity in rats, and that psoralen is the hepatotoxic agent in buguzhi (Yu et al., 2019).

Mammalian target of rapamycin complex 1 (mTORC1) appropriately regulates cell metabolism, proliferation, and cell cycle progression. AKT and adenosine monophosphate-activated protein kinase (AMPK) are important upstream regulatory factors of mTORC1. AKT indirectly activates mTORC1. Conversely, AMPK can inhibit mTORC1. 4EBP1 and p70S6K are the principal signaling molecules downstream of mTORC1 (Liu and Sabatini, 2020). Zhou et al. found that inhibition of mTOR signaling induced by psoralen, which causes activation of AMPK and inhibition of AKT and p70S6K, may also cause S-phase arrest in L02 cells. In addition, psoralen also caused mitochondrial damage, decreased liver regeneration and compensatory capacity, and induced liver damage (Zhou et al., 2018).

In the cell cycle process, activation of cyclin-dependent kinase 2 (CDK2)-cyclin E complexes promotes cells entering into S phase, and then cyclin E is rapidly degraded. At this stage, the gradually increasing cyclin A2 combines with CDK2 to form an activated cyclin A-CDK2 complex, maintaining the progress of S phase and promoting the initiation of DNA synthesis (Hustedt and Durocher, 2016; Du Toit, 2020). For the first time, Zhou et al. found that psoralen-induced S-phase arrest was chiefly related to the up-regulation of p27 and cyclin E1 and down-regulation of cyclin A2. It has been speculated that excessive cyclin E1 may occupy the CDK2 binding site, and then possibly inhibit an association between cyclin A and CDK2, resulting in S-phase arrest (Zhou et al., 2018).

The liver is the principal organ for biotransformation. The cytochrome P450 (CYP450) superfamily is the principal enzyme system in the liver (Kwon et al., 2020). It is generally agreed that the majority of chemicals require metabolic activation prior to the manifestation of toxic effects. Furan ring structures are active functional groups that produce reactive metabolites through CYP450s. A furan ring double bond is oxidized to produce a reactive furanoepoxide or γ-ketoenal intermediate, resulting in irreversible inhibition of CYP450s. A number of active furan epoxides have been demonstrated to cause hepatotoxicity (Mays et al., 1990; Koenigs and Trager, 1998a; Koenigs and Trager, 1998b; Wang et al., 2012; Lu et al., 2016). Song et al. found that psoralen may induce liver injury in rats through the cytochrome P450 metabolic pathway of xenobiotics, among which Akr7a3, Gstm1, Cyp1a2, and Cyp1a1 are important genes in hepatotoxicity, and the endoplasmic reticulum is the principal target subcellular structure. It has been suggested that various cancers and metabolic conditions might be susceptible to hepatotoxicity induced by psoralen (Song et al., 2019). Wang et al. demonstrated that psoralen inhibited the activity and protein expression of CYP2E1 (Wang et al., 2012). Zhuang et al. reported that the inhibitory effect of psoralen on CYP1A2 production was reversible (Zhuang et al., 2013). However, other studies found that psoralen exhibited mechanism-based inactivation (also called irreversible inactivation) of CYP2A6 (Koenigs and Trager, 1998a). and CYP2B1 (Koenigs and Trager, 1998b), in addition to CYP2B6 (Ji et al., 2015) and CYP3A4 (Liu and Flynn, 2015; Hai et al., 2017), which were oxidized by psoralen to produce the reactive metabolite furanoepoxide, suggesting that the metabolite exerted an inhibitory effect rather than the parent compound. In addition, Hai et al. found that the active metabolites can be inactivated by H_2_O and GSH in the liver, helpful for the safe intake of fruits and vegetables containing psoralen, to a certain extent (Hai et al., 2017).

Isoleucine, leucine and valine are standard amino acids with aliphatic side-chains, also known as branched-chain amino acids (BCAAs). There have been reports of changes in BCAAs in the diagnosis of liver dysfunction (Rawat et al., 2017). Zhang et al. revealed that psoralen caused increased production of ALT and AST, decreased Glu and ALB, and interference in amino acid metabolism (Zhang et al., 2018).

### Renal Damage

Diabetic nephropathy is the most serious complication of diabetes, causing glomerular fibrosis and renal function damage. However, Seo et al. found that psoralen increased the activity of high glucose-treated mice mesangial MES-13 cells, reduced the expression of pro-apoptosis proteins, cleaved PARP and Bad, and promoted expression of the pro-survival markers phospho-Bad (ser112) and Bcl-2. Additional investigation found that psoralen inhibited the expression of mRAI-1 which is related to fibrosis in membrane cells (Seo et al., 2017). It has been suggested that psoralen may ameliorate renal damage caused by high glucose in diabetic patients.

### Embryotoxicity

Oxidative stress and abnormal energy metabolism during embryogenesis can lead to malformations (Dartel et al., 2014; Kupsco and Schlenk, 2015). Xia e*t al*. found that psoralen up-regulated Keap1 expression, while down-regulating Nrf2 and Mn-Sod expression. In addition, an increase in active oxygen generation and malondialdehyde concentration, and inhibition of superoxide dismutase activity also indicated the presence of oxidative stress and inhibition of antioxidant capacity. In addition, psoralen caused increased expression of apaf-1, puma, p53, caspase-3/9, and decreased expression of Bcl-2. Furthermore, down-regulated expression levels of pparα1, fas, and hmgcra demonstrated psoralen-induced abnormal lipid metabolism, causing zebrafish embryo/larval developmental toxicity, reducing the rate of hatching in zebrafish, diminishing their body length, and significantly increasing the rate of deformity. Because the yolk is the only source of energy during zebrafish embryo development, yolk retention, pericardial edema, fish sting defects, and flexion were observed in zebrafish larvae, in addition to toxic effects to the developing heart, liver, phagocytes, and nervous system (Xia et al., 2018).

The BeWo human placental cell line is derived from choriocarcinoma and is used as a rate-limiting barrier model for drug and nutrient exchange between mother and fetus in the placenta (Hawkins et al., 2018; Zhang et al., 2020). Guo et al. used BeWo cells to study the transport mechanisms of psoralen *in vitro*, and found that psoralen passed through the placental barrier *via* passive diffusion without involving the P-gp transporter and was absorbed well in the BeWo cell line (Guo et al., 2015). This indicates that psoralen may pose a potential risk to pregnant women, causing embryotoxicity.

When providing therapeutic doses, attention should be paid to safety, and indicators of liver and kidney toxicity tested. In addition, the potential risk of psoralen to pregnant women and embryos should be further evaluated to ensure its safe use during pregnancy.

## Future Prospects

Natural products generally have the effect of improving disease through the intestinal flora (Feng et al., 2019). For example, osteoporosis is a highly common disease. One area of future osteoporosis research is the study of human intestinal microbes (Chen Y. et al., 2017). Some animal model studies (Jones et al., 2017) and human studies (Ji et al., 2017) have provided convincing evidence for the importance of gut microbes in bone metabolism and health. However, there are no literature reports on the effect of psoralen on the intestinal flora to prevent and treat osteoporosis, and the specific mechanism requires further study.

## Conclusions

*Cullen corylifolium*(L.) Medik, a traditional Chinese medicine, has been widely used in clinics to treat various diseases such as osteoporosis, lung cancer, osteoarthritis, *etc*. In the present review, the effects of psoralen, the active ingredient of *Cullen corylifolium*(L.) Medik, were compared using modern scientific methods in order to that its pharmacological consequences can be better understood. We have summarized the results of the most recent pharmacological research, including its use in anti-osteoporosis, anti-tumor, antiviral, antibacterial, anti-inflammatory, photosensitivity, anti-neurodegenerative diseases, anti-depression applications and in liver and kidney toxicity studies. The studies of molecular mechanisms indicate that psoralen regulates osteoblast/osteoclast/chondrocyte differentiation or activation through regulation of the BMP signaling pathway, wnt/β-catenin signaling, IRE1/ASK1/JNK pathway, NF-κB-MAPK pathway, AKT and AP-1 pathways, and levels of miR-488, PPARγ and MMP expression. It also regulates the Wnt/β-catenin signaling pathway to promote the expression of cyclin D1, inducing cell cycle arrest in breast cancer. PERK/ATF4 and ATF6/CHOP-related pathways trigger endoplasmic reticulum stress-mediated apoptosis. Multi-drug resistance can be reversed by inhibition of the activity of P-gp related transporters and enzymes. In addition, psoralen can inhibit DNA viruses, RNA viruses, and bacteria. Interestingly, psoralen may be a potential agent against SARS-CoV-2 infection through the induction of IFN-β secretiom. Furthermore, psoralen inhibits TNF-α, TGF-β, IL-4/5/6/8/13, and Th2 transcription factor GATA-3 protein expression, thus inhibiting the inflammatory response. In addition, psoralen can cause melanin dispersion and inhibit acetylcholinesterase. Finally, research has found that liver and kidney toxicity is caused by its effect on cytochrome enzymes. As shown in this article, psoralen causes a wide range of pharmacological effects, which we believe will attract additional research effort in the future.

## Author Contributions

YR and HL collected literatures. YR, XS, LT, CG, and MW analyzed literatures and summarized results. YR drafted the manuscript. ZC, YL, and CP revised the manuscript.

## Funding

This work was supported by the National Natural Science Foundation of China (grant numbers 81300437, 81403149, 81973189), National Major Scientific and Technological Special Project of China (grant numbers 2017ZX09201001-008), Sichuan Science and Technology Program (grant numbers 2018SZDZX0017), and Scientific Research Fund of CDUTCM (Grants QNXZ2018007, QNXZ2018011).

## Conflict of Interest

The authors declare that the research was conducted in the absence of any commercial or financial relationships that could be construed as a potential conflict of interest.
